# Essential Electronic Properties of Silicon Nanotubes

**DOI:** 10.3390/nano11102475

**Published:** 2021-09-22

**Authors:** Hsin-Yi Liu, Ming-Fa Lin, Jhao-Ying Wu

**Affiliations:** 1 Department of Physics/QTC/Hi-GEM, National Cheng Kung University, Tainan 701, Taiwan; buttid41@gmail.com (H.-Y.L.); mflin@mail.ncku.edu.tw (M.-F.L.); 2 Center of General Studies, National Kaohsiung University of Science and Technology, Kaohsiung 811, Taiwan

**Keywords:** nanomaterials, nanotubes, electronic properties

## Abstract

In this work, the various electronic properties of silicon nanotubes (SiNTs) were investigated by the density functional theory. The cooperative and competitive relationships between the chiral angle, periodic boundary conditions, and multi-orbital hybridizations create unusual narrow gaps and quasi-flat bands in the ultra-small armchair and zigzag tubes, respectively. The features varied dramatically with tube radii. Armchair SiNTs (aSiNTs) have an indirect-to-direct band gap transition as their radius is increased to a particular value, while zigzag SiNTs (zSiNTs) present a metal-semiconductor transition. The projected density of states was used to elucidate the critical transitions, and the evolution of p and s orbital mixing states during the process are discussed in detail. The information presented here provides a better understanding of the essential properties of SiNTs.

## 1. Introduction

Carbon nanotubes (CNTs, single wall) contain a honeycomb lattice with various nanometer diameters and are regarded as a quasi one-dimensional (1D) tubular material [1,2,3,4,5,6,7]. The curvature effect leads to the misorientation of 3$p_{z}$ orbitals and induces significant s$p^{3}$ hybridizations on the cylindrical surface, unlike the pure $sp^{2}$ hybridization of planar graphene. Therefore, CNTs act as metallics or semiconductors depending on their diameter and chiral vector. The large variety of electronic properties in different CNT structures ensure their potential application in many areas [8,9,10,11,12,13,14,15,16,17,18,19,20,21].

Silicon is a group-IV element with a similar electronic configuration to carbon atoms. However, the size of Si compared to C causes it to fill the $sp^{3}$ hybridization state preferentially. Early on, Fagan and Baierle et al. proposed hypothetical silicon nanotubes and studied their electronic and structural properties [22]. They considered two Si nanotube geometry types: hexagonal (h-NTs) and gear-like nanotubes (g-NTs). The two types differ in their Si atomic hybridization, where one is a rolled planar silicene (a monolayer sheet of Si atoms) and the other a buckled silicene that forms a nanotube. At the same time, Si g-NTs are more energetically stable than Si h-NTs [23].

SiNTs were first successfully synthesized in 2001 [24]. Different SiNT growth processes have been reported since then [25,26,27,28,29,30,31]. Sha et al. used a nano channel Al${}_{2}$O${}_{3}$ [26] to fabricate SiNTs by chemical vapor deposition (CVD) [26,32,33]. After that, a variety of techniques have been used to create SiNTs, such as molecular beam epitaxy (MBE) [27], hydrothermal synthesis [29], and gas phase synthesis [30,34]. Ishai and Patolsky demonstrated that the diameter and wall thickness could be controlled between 1.5 to 500 nm [35]. The following are several possible applications for SiNTs. Yoo et al. used a surface sol-gel reaction to obtain SiNTs for energy storage devices [36]. Some other groups enhanced SiNT fabrication techniques to improve their properties for biological, chemical, and electrical applications in sensors, nanoscale electronics, and optoelectronics [37,38,39].

The electronic properties of SiNTs were theoretically studied by using the tight-binding model [23,40,41,42,43,44,45] and the first-principles calculations [22,46,47,48]. Most papers focused on the conducting properties, i.e., the band gap variations, but the detailed geometric parameters, a whole feature of energy bands, spatial charge density, the sophisticated projected density of states (PDOSs), and their dependence on chirality and diameter are still lacking. In this work, we used the first-principles density functional theory (DFT) under the Vienna ab initio Simulation Package (VASP) to thoroughly explore the essential properties of SiNTs. A whole feature of energy bands is discussed, including two groups of σ parabolic bands, frequent anti-crossings between the σ and π bands, a peculiar quasi-flat band in ultra-small zSiNTs near the Fermi level, the initial/final energies of σ and π bands and their mixing strongly dependent on the tube radius and chirality, etc. The above features of energy bands were not presented or discussed well before. Further, we display the spatial charge density distribution to clearly show the transition from multi-orbital to single-orbital hybridizations when increasing the radius. A significant difference in the transition process between armchair and zigzag SiNTs exists. The sophisticated PDOSs reveal the equal and nonequal contributions of $p_{x}$ and $p_{y}$ orbitals to the highest valence band (HVB) edges with $sp/sp^{2}/sp^{3}$ bonding in the indirect-/direct-gap semiconductors for various radii. The above information is helpful to understand the important features in the energy bands, such as the unusual small energy gaps, nonmonotonous energy dispersions, and the existence of quasi-flat and anti-crossing bands.

## 2. Materials and Methods

The first-principles calculations based on the density functional theory (DFT) were carried out using the Vienna ab initio Simulation Package (VASP) [49,50]. The exchange-correlation energy derived from many-particle Coulomb interactions was evaluated using the Perdew–Burke–Ernzerhof functional (PBE) [51] under generalized gradient approximation. The one-dimensional periodic boundary condition was set along the y-direction, and the vacuum distance in the x- and z-space was set to 15 Å to make sure no interaction with neighboring SiNTs. The convergence of the Helmann–Feynman force was less than 0.01 eV/Å during ionic relaxations. The maximal cutoff energy of the wave function expanded by the plane wave was 500 eV. The Brillouin zone (BZ) and geometric optimization were implemented with 1 × 1 × 15 k-point mesh and 1 × 1 × 500 k-point mesh for the PDOSs calculations via the Gamma scheme.

## 3. Results

### 3.1. Geometric Structures

**Figure 1:**
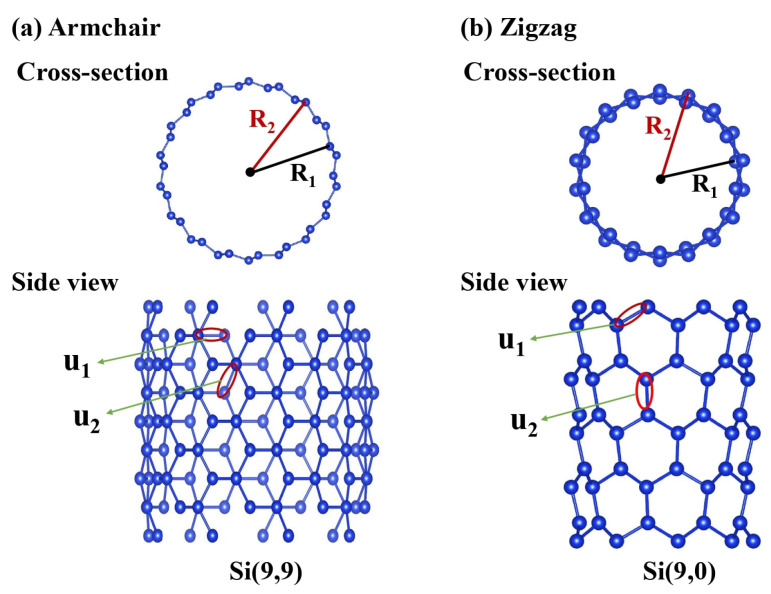
Cross-section and side views for (**a**) armchair (9,9) and (**b**) zigzag (9,0) SiNTs.

SiNTs are akin to rolling up a silicene sheet from the origin to the lattice vector (chiral vector) ${\vec{C}}^{h}$ = n${\vec{a}}_{1}$ + m${\vec{a}}_{2}$ ≡ (n,m), where ${\vec{a}}_{1}$ and ${\vec{a}}_{2}$ are primitive hexagonal lattice vectors. We focus on axial and diagonal chiral vectors, namely armchair (n,n) and zigzag (n,0) types, for which cross-section and side views are presented in Figure 1. The buckling, enhanced by $sp^{3}$ hybridization, results in a gear-like structure on the cylindrical surface. Two inequivalent silicon atoms have smaller and larger distances to the tube axis, $R_{1}$ and $R_{2}$, respectively. The periodic length of the 1D unit cell is $\sqrt{3}u_{2}$ and 3$u_{2}$ for armchair and zigzag, respectively, where $u_{2}$ is the Si-Si bond length “along” the axis.

The optimized geometric parameters, including the optimal lattice constant, the ground state energy $E_{0}$, Si-Si bond lengths $u_{1}$ (“perpendicular” to the axis) and $u_{2}$ (“along” the axis), radii $R_{1}$ and $R_{2}$, and buckling distance Δz, are summarized in Table 1 for the armchair (n,n) and zigzag ($n^{\prime }$,0) tubes with n ∈ (2,25) and $n^{\prime }$∈ (4,21), respectively. The reported radius of the SiNTs was adjustable in the range of 7.5–25 Å using ultra-high vacuum chemical vapor deposition (UHV-CVD) [35]. The following are the results of $R_{1}$($R_{2}$) in the range of 1.731–26.428 (1.826–12.659) and 2.688–26.617 (2.881–13.129) Å for aSiNTs and zSiNTs, respectively. The buckling distance Δz, determined by $R_{2}-R_{1}$, is inversely proportional to the radius, roughly. In addition, the difference between the two bond lengths $u_{1}$ and $u_{2}$ (in units of the standard Si-Si bond length, $u_{0}\approx$ 2.28 Å) reflects the geometric distortion. When a silicene sheet is rolled up to form a tube, it could induce stress ($u_{1,2}<1$) and strain ($u_{1,2}>1$) between Si atoms. Table 1 shows more stress in the armchair tubes and strain in zigzag tubes. The ground state energy $E_{0}$ decreases as the radius increases according to the reduction in the curvature energy [52] and buckling.

### 3.2. Energy Bands

**Figure 2:**
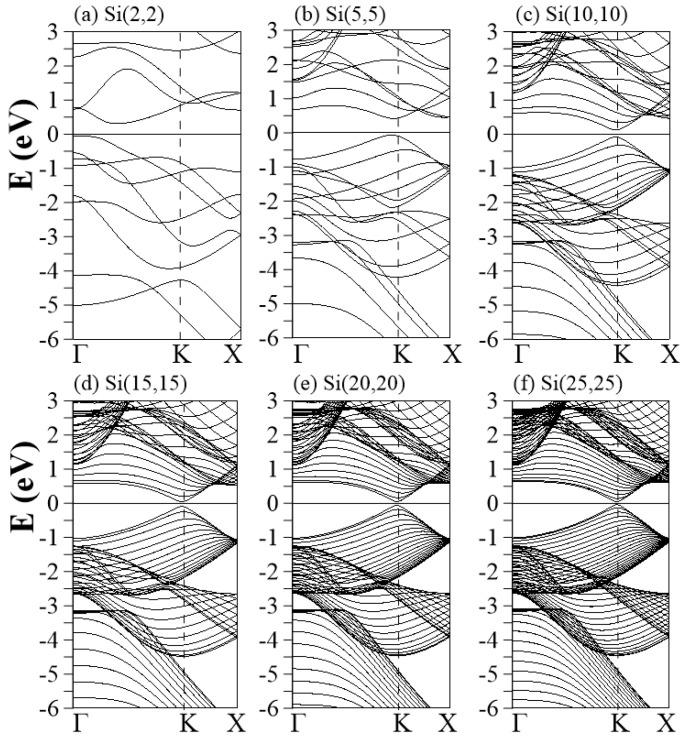
Band structures of aSiNTs with different chiral vectors: (**a**) (2,2), (**b**) (5,5), (**c**) (10,10), (**d**) (15,15), (**e**) (20,20), and (**f**) (25,25).

The aSiNT band structures with chiral vectors of (2,2), (5,5), (10,10), (15,15), (20,20), and (25,25) are shown in Figure 2a–f. There are two high-symmetry points in the 1D BZ of the nanotube, namely, the Γ point at k = 0 and X point at k=±π/a, where a is the lattice constant of the nanotubes. Another crucial point is located between Γ and X, namely K at k = 2π/3a. According to the BZ folding scheme, the Dirac cone at (2π/3a, 2$\pi /\sqrt{3}$a) in the 2D BZ of hexagonal monolayers is folded into the K point (k = 2π/3a) of the 1D BZ [53,54,55]. Therefore, the Γ and K points significantly influence the magnitude of band gaps. Unlike the metallic armchair CNTs, semiconducting behaviors are found in all aSiNTs. The low-energy bands of ultra-small aSiNTs are strongly dependent on the radius (Figure 2a–c). Si(2,2) presents an indirect energy gap ($E_{g})$ of about 0.35 eV. The HVB at point Γ is associated with the misorientation of the 2$p_{z}$ orbitals, which induces hybridization of the p and s states. On the other hand, the lowest conduction band (LCB) is between points Γ and K. The conduction and valence bands are asymmetric to $E_{F}$ = 0 due to low symmetry in the geometric structure (the deformed honeycomb structure). The large curvature suppresses the π and $\pi^{*}$ bands. Accordingly, it is impossible to use single-orbital bonding (2$p_{z}$ orbital) to investigate the essential low-energy properties of the ultra-small tubes. When the radius increases, a valence and a conduction π band approach the Fermi level, leading to a direct gap around the K point (with a slight red shift with respect to the exact K point). The low-energy bands (15,15)–(25,25) have similar features, including a tiny direct gap close to the exact K point and the symmetric π and $\pi^{*}$ bands against the $E_{F}$ = 0, as shown in Figure 2d–f. Two groups of σ parabolic bands are initiated at the Γ point ranging from $E^{v}=$−1.25 to −2.7 eV, associated with the σ-bonds between the neighboring $p_{x}$ and $p_{y}$ orbitals, respectively. Their non-degenerate energies arise from the different boundary conditions along the circumference and tube axis. The deep initial energy of the σ bands (≈−1.25 eV) reveals strong p-p bondings on a relatively flat surface. These properties contrast with the ultra-small tubes, e.g., Si(2,2), where the weak p-s bonding states have almost zero energy. The σ bands have frequent anti-crossings with the π bands in the $E^{v}=-2∼-2.5$ eV region, where a lot of extra band-edge states and some quasi partial flat bands exist. This area may have exceeding van Hove singularities (discussed later). The anti-crossing behaviors denote that the buckling and curvature cause complex orbital hybridizations. The s bands, associated with the 3 s orbitals, come into existence at a deeper energy ($E^{v}<$−3 eV) due to the largest ionization energy (the lowest one-site energy in the tight-binding model [56]).

**Figure 3:**
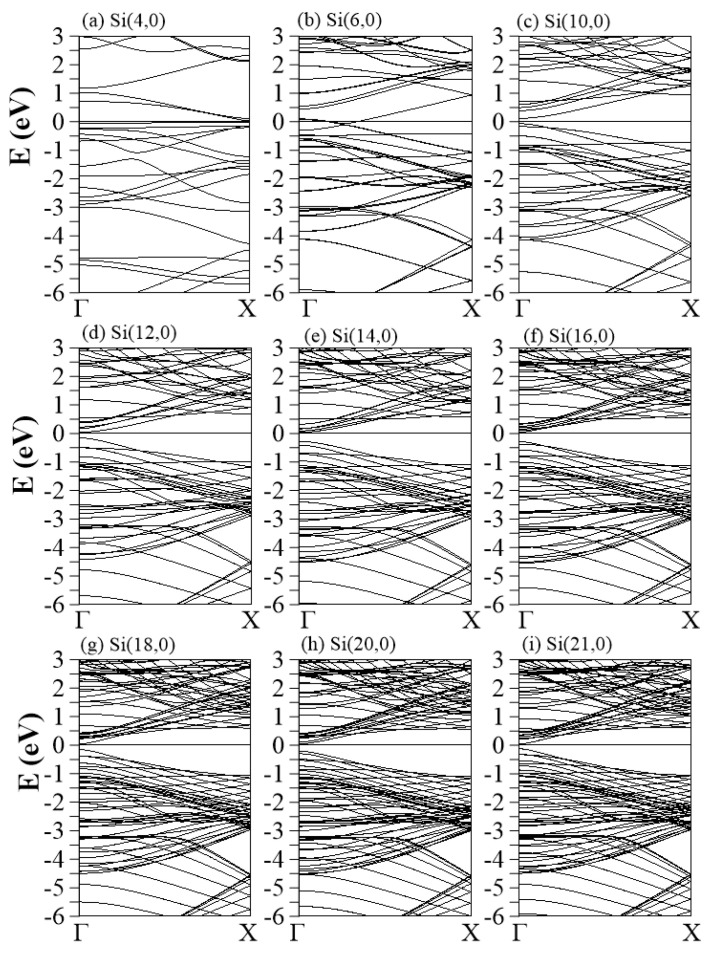
Band structures of zSiNTs with different chiral vectors: (**a**) (4,0), (**b**) (6,0), (**c**) (10,0), (**d**) (12,0), (**e**) (14,0), (**f**) (16,0), (**g**) (18,0), (**h**) (20,0), and (**i**) (21,0).

**Figure 4:**
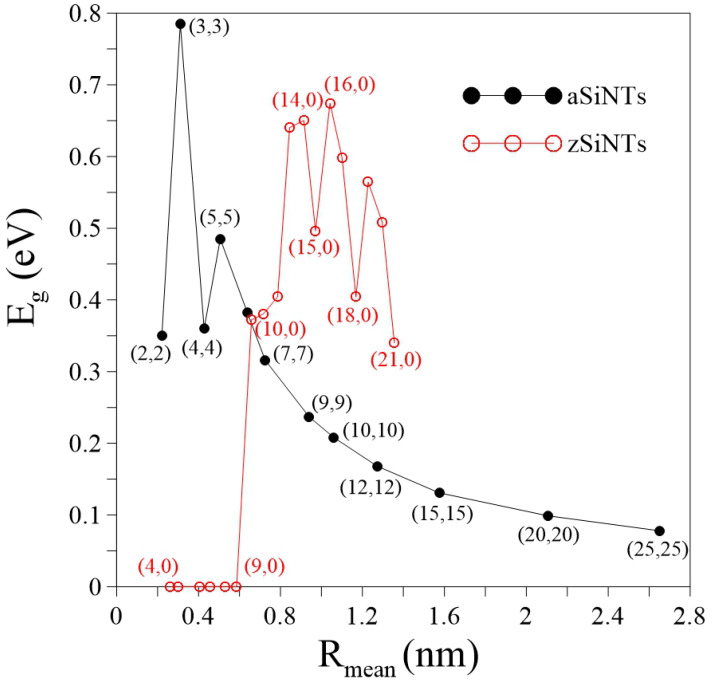
The dependence of band gap $E_{g}$ on the radius for aSiNTs (black curve) and zSiNTs (red curve).

The zSiNTs energy dispersions of Si(4,0)–Si(21,0) are shown in Figure 3a–i. The zSiNTs present a wider variety of low-energy properties than the aSiNTs when changing the radius. The chiral indexes (n,0) for n = 4–9 exhibit metallic features, while n = 10–21 are semiconductors [45,57,58,59]. In ultra-small zSiNTs, e.g., Si(4,0), there are two quasi-flat bands located between $E^{v}=0∼-0.25$ eV. The dispersionless feature is attributed to the lengthened Si-Si bonds in the strongly distorted honeycomb structure (with additional bondings inside). The HVB (LCB) in Si(6,0) is upward (downward) parabolic dispersive along the Γ-X direction crossing $E_{F}$ = 0 between points Γ and K. The 1D band crossing denotes the metallicity. When the radius is increased, the band crossing is replaced by a tiny band gap at the Γ point, as shown in Si(10,0)–Si(21,0) (the band gap size ($E_{g}$) is discussed later and summarized in Table 1 and illustrated in Figure 4). Only very small zSiNTs belong to the 1D metals. This unusual metallic behavior is not dominated by the periodic boundary conditions and π bonding; it mainly comes from the significant $sp^{3}$-orbital hybridization on a highly curved and buckled surface. The σ and π band mixing may also play a significant role in curved silicene nanoribbons. These materials are in contrast to planar systems, such as graphene, where the σ and π orbitals appear orthogonal and cannot exist in a mixed state [60,61]. It is worth noting that a peculiar quasi-flat band exists in all Si(n = even,0) tubes that moves from $E^{v}\approx -0.25$ eV (Si(4,0)) to $E^{v}\approx -1$ eV (for Si(n>10,0)). The appearance or absence of the quasi-flat band in Si(n = even/odd,0) tubes are related to the D${}_{nh}$/D${}_{nd}$ symmetry [56].

### 3.3. Band Gap Variation with Tube Radius

The size of the band gap ($E_{g}$) versus the tube mean radius ($R_{mean}=\frac{R_{1}+R_{2}}{2}$) deserves further discussion (Figure 4). The indirect $E_{g}$ increases and decreases as the radius increases for Si(2,2)–Si(4,4). The band gap size variance occurs according to the movement of the HVB-edge state from the Γ to K points. $E_{g}$ increases again when the direct $E_{g}$ is formed in Si(5,5). After that, $E_{g}$ decreases monotonically as the radius increases further. The narrow gaps induced in aSiNTs are attributed to the combined effects of buckling and curvature, which considerably contrasts with metallic aCNTs [62]. The buckling (summarized in Table 1) and curvature decrease as the radius increases, causing a reduction in $E_{g}$. The zigzag tube zero gaps in Si(4,0)–Si(9,0) correspond to the existence of quasi-flat bands or 1D band crossings. Non-zero $E_{g}$ occurs in Si(10,0) and reaches the first local maximum in Si(14,0). After that, $E_{g}$ presents an oscillatory behavior that includes the global maximum in Si(16,0) and the local minima (nonzero) in Si(15,0), Si(18,0), and Si(21,0), i.e., when n = 3q (q is an integer) [48]. Some publications discussed hexagonal zSiNTs (in analogy to carbon nanotubes) [23,45]. In this condition, n varied from 5 to 12 are metals. Band gap is opened at n = 13 and then presents an oscillatory behavior with the zero gaps occurring at the chiral vector (n = 3q,0). That is, the gear-like zSiNTs with large radii (n = 10–21) are always semiconductors, while the hexagonal zSiNTs have similar electronic properties with carbon nanotubes, which can be metal or semiconductors. The semiconducting features in the gear-like zSiNTs may be attributed to the two inequivalent silicon atoms that have the different distances to the tube axis (Table 1).

### 3.4. Spatial Charge Density Distribution

**Figure 5:**
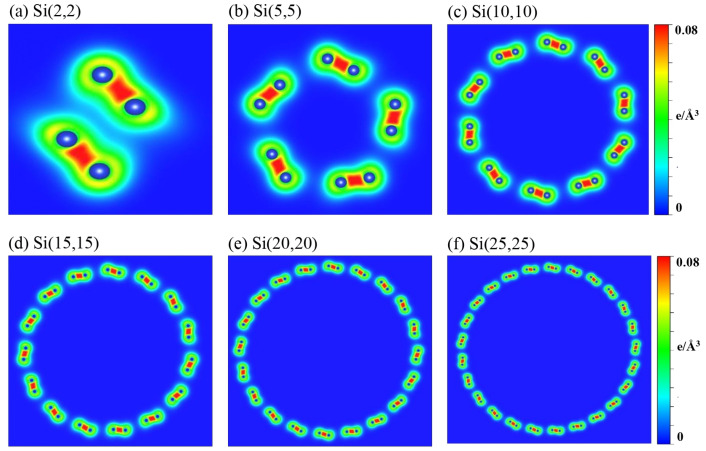
The spatial charge densities on the (x,z)-plane for aSiNTs with different chiral vectors: (**a**) (2,2), (**b**) (5,5), (**c**) (10,10), (**d**) (15,15), (**e**) (20,20), and (**f**) (25,25).

**Figure 6:**
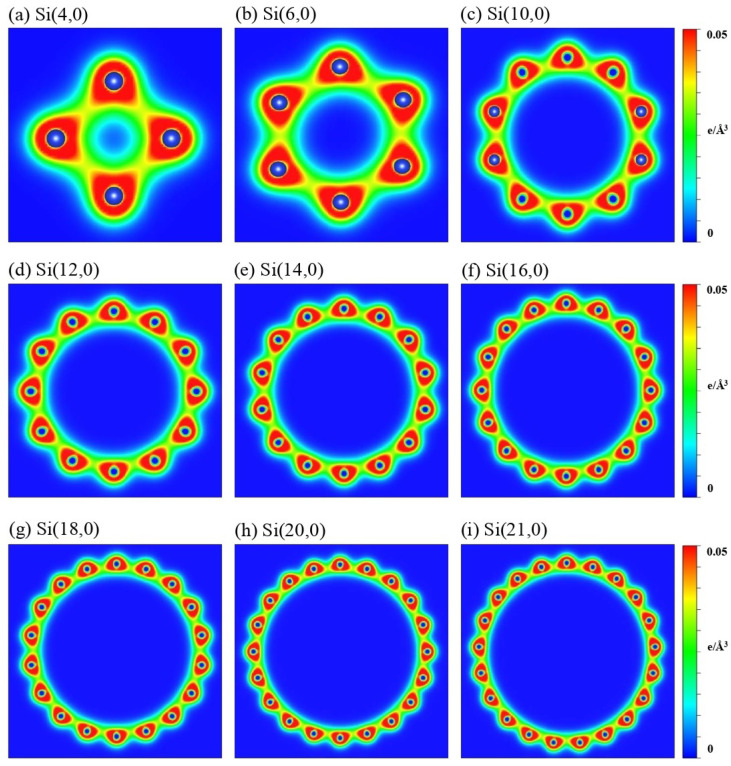
The spatial charge densities on the (x,z)-plane for zSiNTs with different chiral vectors: (**a**) (4,0), (**b**) (6,0), (**c**) (10,0), (**d**) (12,0), (**e**) (14,0), (**f**) (16,0), (**g**) (18,0), (**h**) (20,0), and (**i**) (21,0).

The low-lying energy bands are closely related to the type of orbital bonding. Spatial charge distributions, which are sensitive to the surface profile, are used for identifying and examining the multi- and single-orbital hybridizations and the charge transfer [63]. Armchair and zizag SiNTs present similar and distinct charge distributions, as do the orbital hybridizations, demonstrated by the (x,z)-plane projection shown in Figure 5 and Figure 6, respectively. The aSiNTs possess clear σ and π bonds (Figure 5). The (3 s, 3$p_{x}$, 3$p_{y}$) orbitals of Si interact with adjacent Si through σ bonds (red rectangles), while the perpendicular 2$p_{z}$ orbitals show significant π bonding (green rectangles). Both bond types survive in the ultra-small tubes but become non-orthogonal to each other. The σ bond is strengthened with the truncated bond length as the radius increases and the buckling decreases, while the opposite is true for the observable sp${}^{3}$-orbital bonds. With a sufficiently large radius, e.g., Si(15,15)–Si(25,25), the σ-bond strength is similar to planar monolayer silicene [64], i.e., the tubular structure does not affect the charge distribution much, as revealed in the bond lengths $u_{1}$ and $u_{2}$ that are close to 1 (Table 1). The significant $sp^{3}$-orbital bonds in the small tubes result from the misorientations in the (3$p_{x}$, 3$p_{y}$, 3$p_{z}$) orbitals. The misorientations lead to drastic changes in the low-energy band structures, e.g., the unusual small energy gaps and the nonmonotonous energy dispersions (Figure 2 and Figure 3). To fully comprehend the essential properties of the gear-like tubular structures, non-negligible sp${}^{3}$ multi-orbital hybridizations might be simultaneously included in the tight-binding model. These four-orbital hybridizations are more pronounced in zigzag than armchair systems (Figure 6) due to the longer Si-Si distance along the azimuthal direction. The charge density is more localized around the Si atoms, especially with a small radius, e.g., Si(4,0). The yellow region between the neighboring Si atoms is dominant in Si(4,0), which corresponds to an sp${}^{3}$-orbital bond. The red region (highest density) in the semi-ellipse shape gradually transforms into a triangle as the radius is increased. This change is caused by the decreased buckling that causes significant charge transfer from the sp${}^{3}$-orbital bond to the sp${}^{2}$-orbital bond.

### 3.5. The Projected Density of States

**Figure 7:**
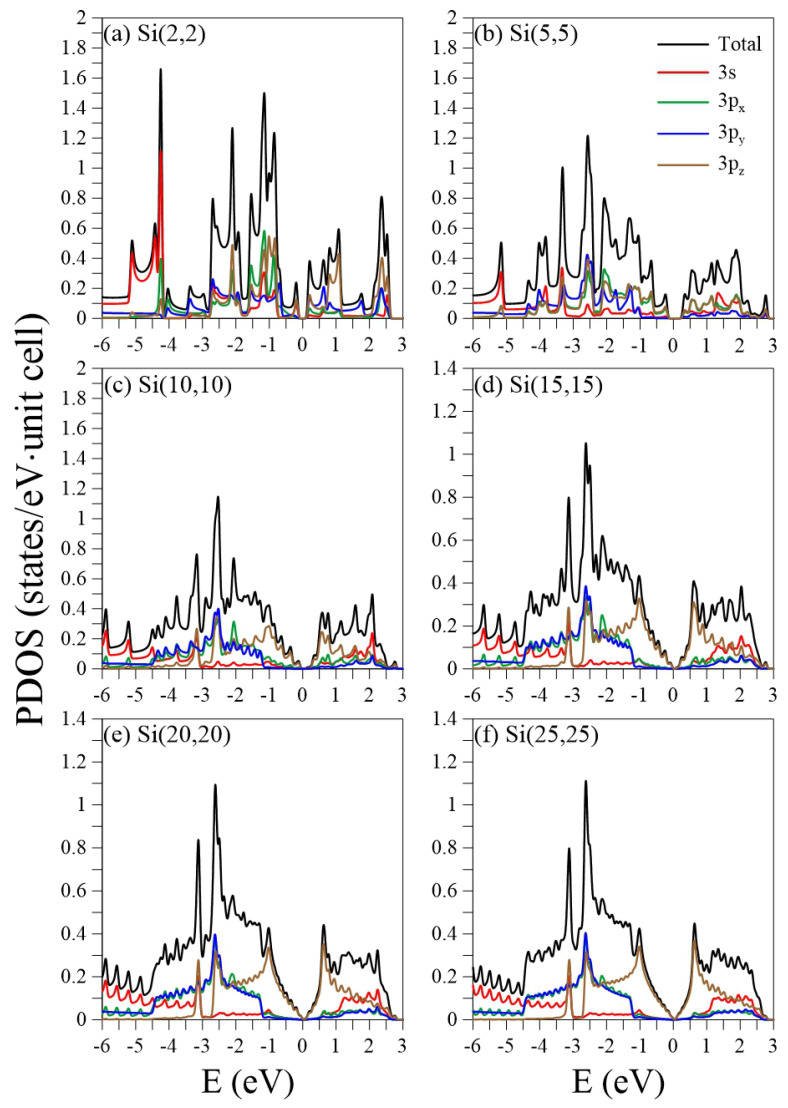
The orbital-projected densities of states for aSiNTs with different chiral vectors: (**a**) (2,2), (**b**) (5,5), (**c**) (10,10), (**d**) (15,15), (**e**) (20,20), and (**f**) (25,25).

**Figure 8:**
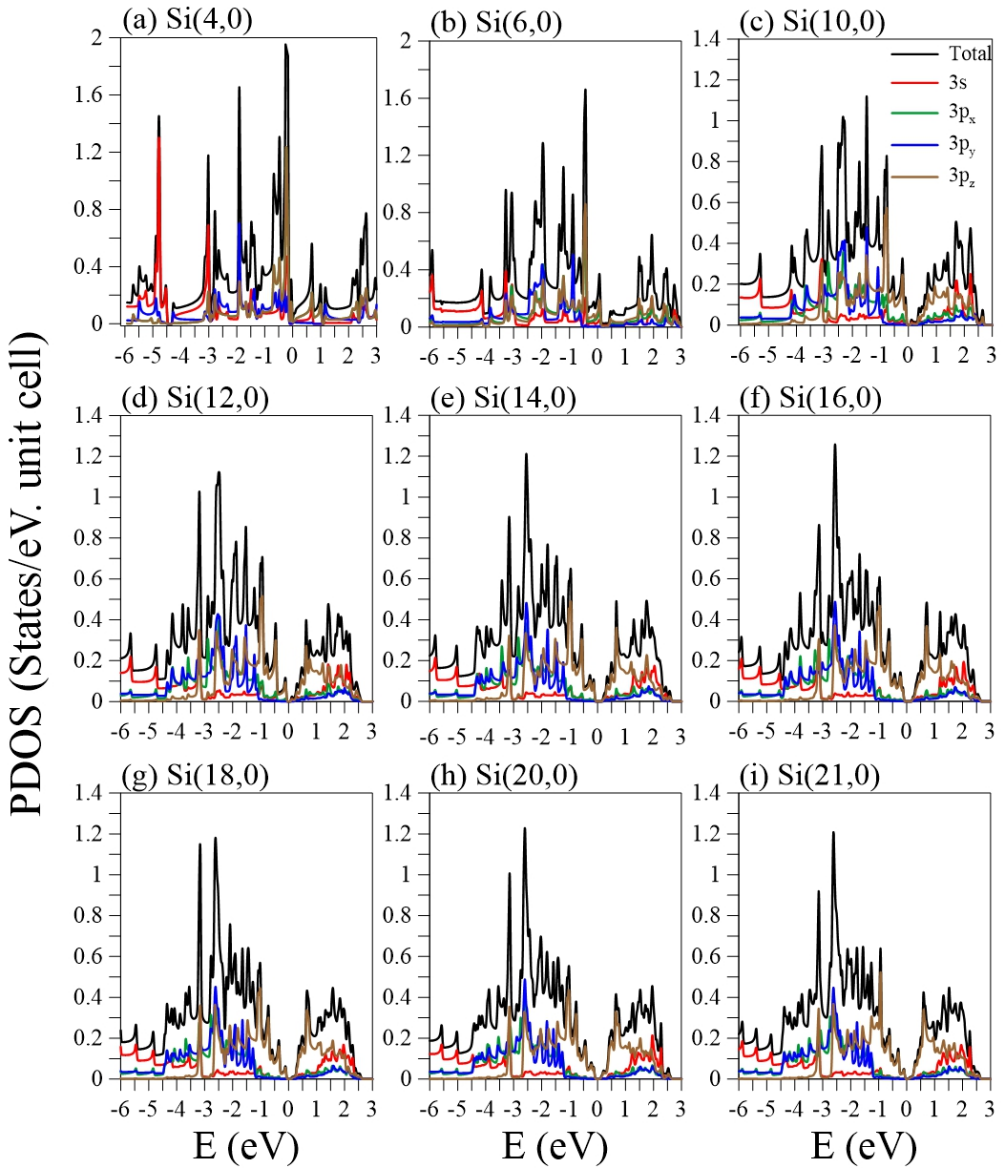
The orbital-projected densities of states for zSiNTs with different chiral vectors: (**a**) (4,0), (**b**) (6,0), (**c**) (10,0), (**d**) (12,0), (**e**) (14,0), (**f**) (16,0), (**g**) (18,0), (**h**) (20,0), and (**i**) (21,0).

The projected density of states (PDOS) provides valuable information about the cooperative and competitive relationships between the chiral angle, the periodic boundary conditions, and the multi-orbital hybridizations, as shown in Figure 7 and Figure 8. Most of the peaks are asymmetric, except for a few symmetric peaks that correspond to quasi-flat bands. A pair of asymmetric peaks in aSiNTs (Figure 7) near the Fermi level is associated with the tiny band gap and low-lying parabolic energy bands (Figure 2). However, their compositions strongly depend on the radius. The HVB edge compositions in the indirect-gap semiconductor Si(2,2) come from the $p_{z}$ (brown curve) and s (red curve) orbitals equally. However, for the direct-gap semiconductor Si(5, 5), the HVB edge compositions come from the $p_{z}$- and $p_{x}$-orbitals (green curve). The $p_{x}$-orbital component gradually decreases as the radius increases. Subsequently, the PDOSs in Si(15,15)–Si(25,25) around the $E_{F}$ = 0 are dominated by the $p_{z}$-orbital only, similar to monolayer silicene. The more significant contribution of $p_{x}$ compared to $p_{y}$ (blue curve) to the HVB edges in small tubes is ascribed to the strong curvature-induced hybridization along the circumference direction. For large tubes, e.g., Si(10,10)–Si(25,25), the $p_{z}$-orbital begins at the HVB edge (close to the zero energy), while the $p_{x}$ and $p_{y}$ orbitals start at E${}^{v}$≈−1.2 eV. The former and latter have a strong hybridization at E${}^{v}\approx$−2.5 eV, where the most substantial peaks exist. Many anti-crossing bands occur at this energy (Figure 2c–f). Though the compositions of the HVB edges in zSiNTs with small radii are similar to aSiNTs, the PDOS has a finite value at $E_{F}$ = 0, e.g., in Si(4,0)–Si(6,0), reflecting metallic behavior due to the quasi-flat bands (Figure 3a) and the 1D band crossings (Figure 3b). The similarities in PDOS features between aSiNTs and zSiNTs include a strong dominance of the π-orbital around $E_{F}$ = 0 in large raddi conditions and the initial energy of $E^{v}\approx$−1.2 eV for the $p_{x}$ and $p_{y}$ orbitals. On the other hand, large aSiNTs, e.g., Si(15,15), Si(20,20), and Si(25,25) show two strong symmetric peaks at E${}^{c}\approx$−1 and −3.2 eV. The former corresponds to the highly degenerate states at the X point, while the latter corresponds to the quasi-flat band induced by the $sp^{3}$-orbital hybridization. Only the latter one exists in zSiNTs. Generally, independent 3$p_{z}$ orbitals are not observable in the small armchair or zigzag SiNTs, but the opposite is true in large ones. In the large SiNTs, the π-orbital is in the range of $E^{v}=0∼-1$ eV, similar to monolayer silicene.

## 4. Conclusions

In summary, the geometric structure and electronic properties of SiNTs with different chirality and diameters were computed and analyzed in detail using first-principles calculations. The results demonstrate that strong multi-orbital hybridizations induce semiconducting and metallic properties in the small armchair and zigzag tubes, respectively. There is an indirect-to-direct band gap transition in aSiNTs when the tube diameter increases, while a metal-semiconductor transition occurs in zSiNTs. The transitions are attributed to the decreasing curvature and buckling, which transfers the complex orbital hybridization to single-orbital bonding. The orbital hybridization creates various electronic properties, including unusual small energy gaps, nonmonotonous energy dispersions, the existence of quasi-flat bands, and frequent anti-crossings between σ and π bands. The calculated PDOS reveals the equal and nonequal contributions of $p_{x}$ and $p_{y}$ to HVB edges for sp/sp${}^{2}$/sp${}^{3}$ bonding and zero and nonzero PDOS at $E_{F}$ = 0 depending on the chirality and diameters. In addition, a strong symmetric peak located at the middle energy level ($E^{v}$= −3.2 eV) demonstrates the significant sp${}^{3}$ bond. These findings shed light on the potential applications of SiNTs in nanoelectronic devices.

## Figures and Tables

**Table 1:** Geometric properties of aSiNTs and zSiNTs: lattice constant, ground state energy $E_{0}$, Si-Si bond lengths $u_{1}$ (“perpendicular” to the axis) and $u_{2}$ (“along” the axis), radii $R_{1}$ and $R_{2}$, buckling distance Δz, and band gaps $E_{g}$.

aSiNTs	Lattice con. (Å)	$E_{0}$ (eV/atom)	$u_{1}$/$u_{0}$	$u_{2}$/$u_{0}$	$R_{1}$ (Å)	$R_{2}$ (Å)	Δz (Å)	$E_{g}$ (eV)
Si(2,2)	3.54	−4.746	1.003	1.043	1.731	2.688	0.957	0.350
Si(3,3)	3.722	−4.786	1.001	1.015	2.500	3.181	0.681	0.785
Si(4,4)	3.751	−4.812	1.000	1.009	3.967	4.569	0.602	0.360
Si(5,5)	3.764	−4.828	0.999	1.005	4.796	5.325	0.529	0.485
Si(6,6)	3.766	−4.837	0.999	1.003	6.118	6.648	0.530	0.382
Si(7,7)	3.772	−4.843	0.998	1.002	7.001	7.501	0.500	0.316
Si(9,9)	3.775	−4.850	0.997	1.004	9.149	9.649	0.500	0.237
Si(10,10)	3.779	−4.851	0.997	1.004	10.362	10.851	0.489	0.208
Si(12,12)	3.780	−4.854	0.999	1.000	12.488	12.970	0.481	0.168
Si(15,15)	3.777	−4.856	0.997	0.999	15.595	15.909	0.314	0.131
Si(20,20)	3.782	−4.858	0.996	0.999	20.943	21.151	0.208	0.099
Si(25,25)	3.782	−4.859	0.995	0.998	26.428	26.617	0.189	0.078
**zSiNTs**	**Lattice con. (Å)**	$E_{0}$ **(eV/atom)**	$u_{1}$ **/** $u_{0}$	$u_{2}$ **/** $u_{0}$	$R_{1}$ **(Å)**	$R_{2}$ **(Å)**	Δ **z (Å)**	$E_{g}$ **(eV)**
Si(4,0)	3.806	−4.703	1.132	1.033	1.826	2.881	1.055	0
Si(5,0)	3.814	−4.694	1.022	1.025	2.282	3.163	0.881	0
Si(6,0)	3.757	−4.708	1.008	1.011	3.407	4.075	0.668	0
Si(7,0)	3.760	−4.720	1.006	1.008	3.929	4.505	0.576	0
Si(8,0)	3.770	−4.731	1.004	1.007	4.661	5.222	0.561	0
Si(9,0)	3.773	−4.740	1.004	1.006	5.199	5.733	0.534	0
Si(10,0)	3.773	−4.747	1.004	1.005	5.906	6.434	0.528	0.186
Si(11,0)	3.773	−4.751	1.003	1.004	6.463	6.973	0.510	0.190
Si(12,0)	3.776	−4.755	1.003	1.004	7.145	7.653	0.508	0.202
Si(13,0)	3.779	−4.758	1.003	1.003	7.711	8.206	0.495	0.320
Si(14,0)	3.776	−4.760	1.002	1.002	8.391	8.883	0.492	0.325
Si(15,0)	3.780	−4.762	1.001	1.002	8.945	9.428	0.483	0.248
Si(16,0)	3.777	−4.764	1.001	1.001	9.621	10.104	0.483	0.237
Si(17,0)	3.779	−4.765	1.001	1.001	10.191	10.669	0.478	0.299
Si(18,0)	3.782	−4.766	1.001	1.002	10.845	11.323	0.478	0.202
Si(19,0)	3.781	−4.767	1.001	1.001	11.426	11.899	0.473	0.282
Si(20,0)	3.780	−4.768	1.001	1.001	12.088	12.564	0.476	0.254
Si(21,0)	3.781	−4.769	1.001	1.001	12.659	13.129	0.470	0.170

## Data Availability

The data presented in this study are available on request from the corresponding author.
